# Bioinspired Whisker Sensor for 3D Mapping of Underground Mining Environments

**DOI:** 10.3390/biomimetics9020083

**Published:** 2024-01-31

**Authors:** Virgilio Gomez, Walid Remmas, Miguel Hernando, Asko Ristolainen, Claudio Rossi

**Affiliations:** 1 Centre for Automation and Robotics, Universidad Politécnica de Madrid—CSIC, 28006 Madrid, Spain; miguel.hernando@upm.es (M.H.); claudio.rossi@upm.es (C.R.); 2 Centre for Biorobotics, Department of Computer Systems, School of Information Technologies, Tallinn University of Technology, 12618 Tallinn, Estonia; walid.remmas@taltech.ee (W.R.); asko.ristolainen@taltech.ee (A.R.)

**Keywords:** whisker sensor, 3D mapping, bioinspired design

## Abstract

Traversing through challenging, unstructured environments, particularly in mining scenarios characterized by dust concentration, darkness, and lack of communication presents formidable obstacles for traditional sensing technologies. Drawing inspiration from naked mole rats, characterized as being skilled subterranean navigators that depend heavily on touch to navigate their environment, this study introduces a new whisker-sensing disk designed for 3D mapping in unstructured environments. The disk comprises a circular array of 32 whisker sensors, each featuring a slender flexible plastic rod attached to a compliant base housing a 3D Hall-effect sensor. The whisker sensor is modeled using both analytical and data-driven approaches to predict rotation angles based on magnetic field measurements. The validation and comparison of both models are performed by evaluating data from other whisker sensors. Additionally, a series of experiments demonstrates the whisker disk’s capability in performing 3D-mapping tasks, along with successful implementation on diverse robotic platforms, highlighting its future potential for effective 3D mapping in complex and unstructured subterranean environments.

## 1. Introduction

Navigating unstructured environments poses formidable challenges for autonomous robots, as they struggle with complexities, such as clutter, unpredictable terrain, and the limitations of traditional mapping technologies. This is particularly prevalent in mining environments, known for their extreme and unstructured nature, where dust concentrations and darkness create additional hurdles for traditional localization, sensing, and mapping methods that rely on Global Positioning Systems (GPS), cameras, and Light Detection and Ranging (LiDAR) systems [1]. Recognizing the need for innovative solutions, the development of alternative sensory approaches becomes critical for robots tasked with mapping and navigating these cluttered and difficult-to-access environments [2]. A promising approach involves the use of touch-based sensors to map the surface of the mine, with bioinspired mammalian sensors emerging as a viable alternative due to their demonstrated robustness, simplicity, and precision [3]. The versatility of such sensors is evident in their application for various purposes, ranging from object localization [4,5,6], contour and texture recognition [3,7,8], and even fluid flow direction and viscosity [9,10,11]. With particular emphasis on robot navigation and mapping, the integration of active and passive whiskers into the structure of mobile robots and robot manipulators [12,13,14,15,16] has shown great potential for obstacle avoidance and Simultaneous Localization and Mapping (SLAM) tasks [17,18]. However, not much effort has been put into point cloud generation for environment reconstruction. Mapping 3D spaces presents additional complications, as whisker-based sensors need to navigate and interpret spatial information effectively. Factors such as environmental variability, surface textures, and the need for real-time adaptability contribute to the intricacies faced in developing robust models for whisker-based sensors in 3D-mapping applications.

**Figure 1:**
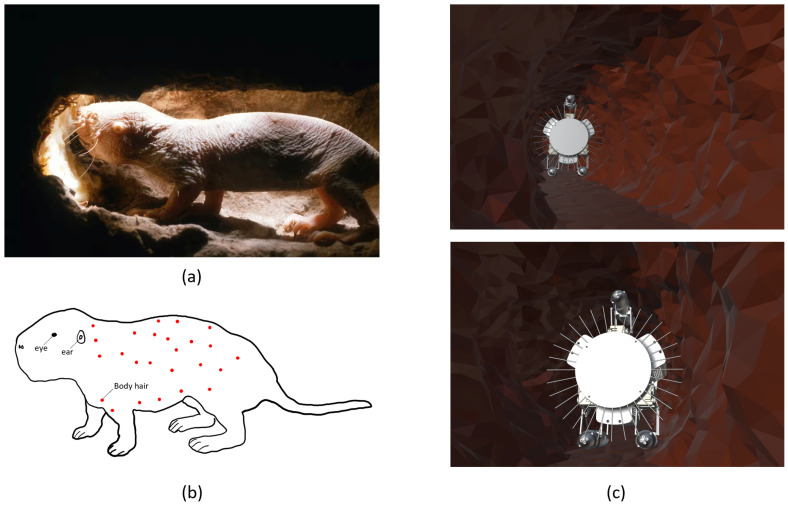
(**a**) Naked mole rat in subterranean environments (Picture taken by Neil Bromhall, Shutterstock). (**b**) Body hair location for an adult naked mole rat. Each body hair is represented by a small red dot. This rodent presents a systematic array of sensitive sensory hairs on its body, as described in [23], that help it navigate and orient itself in lightless subterranean environments. (**c**) Concept of robotic miner equipped with touch based sensor for navigation and mapping of unstructured 3D environments.

The ROBOMINERS project [1] strategically focuses on introducing innovative robotic concepts to overcome these challenges and improve mineral access in Europe, thus reshaping the mining ecosystem. A crucial objective is to enable robots to navigate and execute selective mining procedures by equipping them with the capability to map mining environments and dynamically adjust their paths for optimized mineral extraction. To address this challenge, prototypes that incorporate various alternative sensor technologies are currently in development and are undergoing testing. These prototypes explore a spectrum of options, from spectrometry-based sensors [19] to contact-based sensors. Drawing inspiration from the remarkable tactile abilities of the naked mole rat, a notable rodent species renowned for its navigation and contact detection capabilities in subterranean environments (Figure 1a) [20], an autonomous robotic miner (Figure 1c) could navigate challenging environments, addressing complex issues such as executing mining procedures and precise 3D-mapping tasks and navigating in subterranean areas [21]. Similar to the naked mole rat, which is heavily dependent on its somatosensory systems (touch) [22] and uses a series of sensitive sensory hairs to orient itself and navigate subterranean landscapes (Figure 1b), the robotic miner strives to replicate the efficiency of nature in overcoming obstacles in its quest for optimal performance and adaptability.

This study introduces a novel whisker-based disk that uses 3D Hall-effect sensors to map 3D unstructured environments. The disk design tries to approximate a section of the mole rat body, where the sensory hairs are approximately distributed in a ring-like pattern across the whole body. Compared to other designs, the whisker is housed in a non-permanent manner to the silicone rings, and the magnet holder features a spherical joint that serves as the pivot point to transmit the rotational movement of the whisker. The whisker is modeled following both analytical and data-driven approaches to determine the rotational angles of the whiskers, given the magnetic field felt by the sensor when the whisker makes contact with an object. In this paper, the whisker disk is installed on a serial manipulator to demonstrate the mapping of 3D surfaces. Furthermore, the design can be attached to a scaled-down robotic miner to give the robot the capability to map 3D environments during operation.

This article is structured as follows. In Section 2, we introduce the design and manufacturing process of the sensor. In Section 3, we present an approximation of an analytical model of the phenomena and propose an alternative sensor model based on a data-driven approach. In Section 4, we perform different experiments to compare and validate the rotational models presented. In Section 5, we carry out 3D-mapping experiments using the serial manipulator and showcase the adaptability of the presented solution to other robotic platforms. Finally, in Section 6, we draw conclusions from the results obtained in the study.

## 2. Sensor Design and Manufacture Process

**Figure 2:**
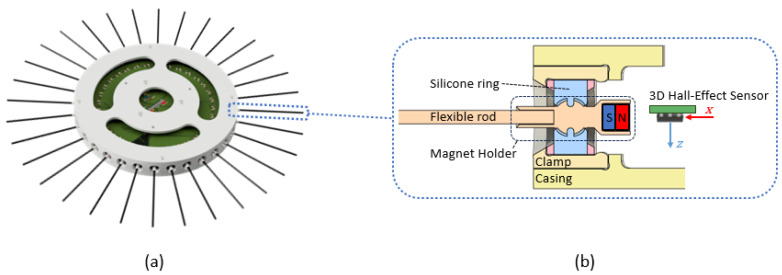
Whisker disk prototype. (**a**) The sensor comprises a circular array of 32 whiskers. (**b**) Detailed section view of a single whisker sensor.

The prototype presented (Figure 2a) is based on a circular array of 32 whiskers that can be attached to different robots to map non-structured environments. The main component of the disk is the whisker sensor, composed of a slender flexible polystyrene rod that is inserted at one end into a 3D-printed part called a “magnet holder”, where a neodymium permanent magnet is affixed to the opposite end of this piece. The magnet possesses axial magnetization, aligning its magnetic field with the Hall effect sensor’s x-axis. The magnet holder features a spherical contact that is housed inside a silicone structure composed of two rings that are attached to the disk, as shown in Figure 2b. The dimensions of the sphere are slightly larger with respect to the silicone structure to ensure the restriction of the magnet holder inside the rings. The silicone structure allows the magnet holder to rotate in two planes as the whisker makes contact with a surface.

### Sensor Manufacturing Process

The whisker disk pieces were manufactured using a combination of fused deposition modeling (FDM) and stereolithography (SLA) printing. The disk structure was printed using an industrial grade FDM printer (Stratasys, Dimension Elite), while the molds for the inner cavity of the silicone structure and the magnet holder were printed with a high resolution SLA printer (Formlabs Form 3B) with a layer thickness of 50 μm.

To manufacture the silicone rings, we first prepared the mold pieces by applying a silicone release agent and secured them with a set of screws. During the mixing procedure, the silicone mixture (Zhermack Elite double 22) underwent a two-minute vacuum treatment to eliminate any potential bubble formation. Following degassing, the mixture was carefully injected into the mold. Finally, to guarantee the absence of bubbles within the silicone structure, the mold was reintroduced into the vacuum chamber and pressurized throughout the rest of the curing process. This step was repeated for the upper ring, ensuring uniformity and structural integrity.

**Figure 3:**
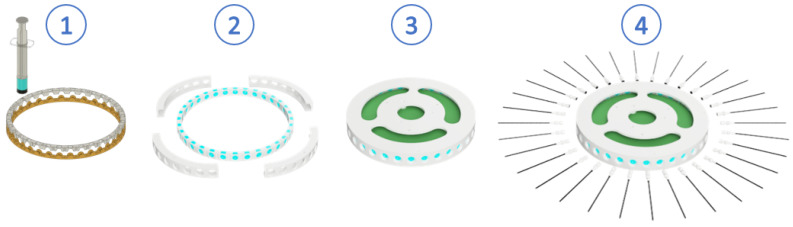
Whisker disk assembly procedure. First, the silicone base is prepared by injecting the mixture into the plastic rings. Next, the silicone rings are clamped and assembled into the disk structure with a set of screws. Finally, the PCB is installed, and the whiskers are inserted through the silicone ring until the spherical contact reaches its position.

Finally, to assemble the whisker disk (Figure 3), we first clamped the two rings with their respective silicone structure and secured the structure inside the casing that comprises the disk with a set of screws. To assemble the magnet holder, we glued the magnets to the rear part and attached the rods to the front part. The magnets used were N52 grade 4mm cylindrical neodymium magnets. The rods selected were 2 mm polystyrene rods. The magnet holder was then inserted into the silicone rings until the spherical housing occupied the silicone cavity. Lastly, a Printed Circuit Board (PCB) disk was installed where the Hall-effect sensors are mounted, ensuring that the corresponding sensor’s X-axis was collinear to each whisker. It should be noted that since the presented prototype is made up of 32 whiskers and the selected Hall-effect sensors do not have enough configurable addresses, it was necessary to include four Inter-Integrated Circuit (I2C) multiplexors (PCA9548AD) in the PCB.

## 3. Sensor Modeling

In order to determine the sensor model that correctly maps sensor measurements, $(B_{x}$, $B_{y}$, $B_{z})$ to rotation angles $(\theta_{y}$, $\theta_{z})$, we performed a comprehensive modeling procedure employing analytical and data-driven methodologies.

**Figure 4:**
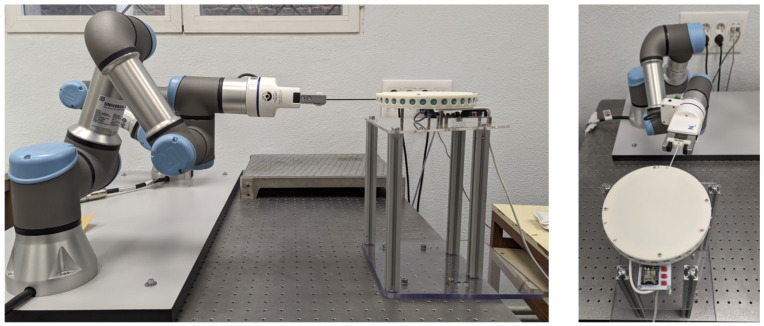
Testbed to capture whisker sensor data featuring a UR3e serial manipulator.

To collect the necessary data to train and validate both models, we designed and built a test platform featuring a Universal Robot (UR) UR3e manipulator. The manipulator gripped a single whisker at its tip and performed randomized movements within its workspace. The setup is presented in Figure 4, where the sequence of movements is based on performing pure rotations with respect to the pivot point of the whisker for both angles. Throughout this process, both the robot’s rotation angles with respect to the whisker and the sensor measurements for each position were recorded. A demonstration of the data capture process is available in the Supplementary Video S1.

The whisker workspace was determined by establishing rotational limits for both possible angles, followed by generating a meshgrid that encompassed all attainable positions. To facilitate movement to these positions, a Python script was written using UR’s RTDE python client library [24]. To read the sensor output, a microcontroller was utilized by running an Arduino routine that averaged the first 16 measurements and, upon request from the Python script, printed the averaged sensor data.

### 3.1. Whisker Rotation Model

**Figure 5:**
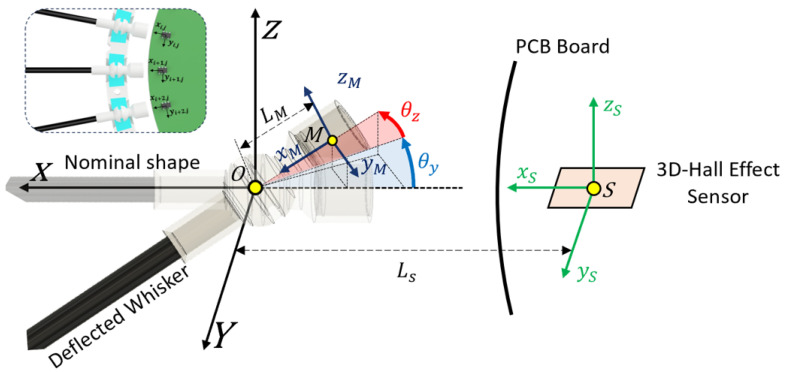
Whisker sensor rotational model description. The whisker can rotate in two different angles from the pivotal point generating the change in the position and orientation of the magnet and therefore of the magnetic field felt by the Hall-effect sensor.

To model the expected rotation of the whisker, given the magnetic field measurements, we considered the relationships provided by the manufacturer to estimate the linear and rotational movements of the magnet, considering an approximation of the relationship between the magnetic fluxes of each axis and the orientation of the magnet [25]. The description of the proposed model and references is presented in Figure 5, where the angles $\theta_{y}$ and $\theta_{z}$ represent the rotation on the y and z axes, *O* represents the pivot point, *M* is the center point of the magnet, and *S* is the center of the sensitive area of the sensor.

The magnet position can be estimated considering the approximation presented in (1–3), where $\vec{B}$ represents the magnetic flux vector measured by the sensor, and $sc_{\theta_{y}}$, $b_{\theta_{z}}$, $b_{\theta_{y}}$ and $b_{\theta_{z}}$ represent the scaling and bias factors for both bending angles, respectively. Both scaling factors and biases are adjusted based on experimental data. It is worth noting that both the earth’s magnetic field and neighboring magnets can affect the magnetic flux of the sensor, therefore changing the estimation of the magnet position. However, these effects resulted in a relatively small impact and were excluded from the model.

(1) $$\vec{B}=\left\{\begin{array}{ccc} B_{x} & B_{y} & B_{z} \end{array}\right\}$$

(2) $$\theta_{z}=\frac{atan2\left(B_{y},B_{x}\right)}{sc_{\theta_{z}}}-b_{\theta_{z}}$$

(3) $$\theta_{y}=-\frac{atan2\left(B_{z},\sqrt{B_{x}^{2}+B_{y}^{2}}\right)}{sc_{\theta_{y}}}-b_{\theta_{y}}$$

Once the rotation angles are calculated, assuming that the point of contact between the whisker and the surface occurs at the tip of the whisker, we can determine an approximate location of the surface point. It should be noted that the error resulting from this consideration is acceptable for a mining scenario, where the whisker length with respect to the overall size of the robot is relatively small.

#### Data-Driven Approach

We decided to implement a data-driven approach based on machine learning algorithms to account for the nonlinearity, variability, and inconsistencies derived from the whisker sensor manufacturing process. This approach allows us to provide a more adaptable solution, with the drawback of reducing the explainability of the model.

To select the algorithm that best combines computational cost and accuracy, we carried out a comparative study. Four state-of-the-art algorithms, Extreme Learning Machine (ELM), Multilayer Perceptron (MLP), Support Vector Regressor (SVR), and K-Nearest Neighbors Regressor (KNN-R) were considered based on previous experience. ELM and MLP are both neural network architectures, but ELM is known for its simplicity and rapid training [26], while MLP typically involves deeper architectures and backpropagation [27]. SVR is based on the principles of support vector machines, where the model learns the importance of a variable to characterize the relationship between input and output [28]. The aim is to find a hyperplane that best fits the data, considering a margin around the predicted values. KNN-R is a non-neural network method that relies on instance similarity [29].

To compare the algorithms, a sensitivity analysis was performed first to identify the models that perform the best for each algorithm. The prediction time and precision were evaluated using the mean absolute error (MAE) and the root mean square error (RMSE) as the main comparison metrics. A total of 900 points were recorded for a single whisker, where the rotations for both angles varied between −20º and 20º. The data were divided in an 85/15 split for training/testing sets, and the algorithms were run on a 1.8 GHz AMD Ryzen 7 5700U computer with 16 GB RAM, running a Python script.

Observing the results in Table 1, it can be seen that the best balance between the computational cost and precision is obtained by the ELM. The four models are capable of predicting the behavior of the whisker given the sensor input. However, the ELM is ranked the highest in speed and the second in terms of precision, only surpassed by the KNN-R algorithm. Recognizing the importance of a rapid and lightweight model is essential to maintain a satisfactory sensing frequency.

## 4. Results and Discussion

Six whiskers were randomly selected and evaluated in terms of the accuracy and computational cost for both models. The analytical model was fine-tuned with respect to scaling and bias factors through an optimization procedure using the nonlinear least squares method, while the data trained a specific ELM neural network algorithm. For each whisker, a test set of 200 points each was evaluated.

Although both models exhibited highly accurate performance across nearly all whiskers considered, the data-driven approach outperformed the analytical method in terms of efficiency. Analyzing Table 2 and Table 3, we observe that the predictions of the ELM model are not only more precise but also faster than those of the analytical model. This efficiency gain can be explained based on the approximations of the analytical model, where there are no considerations for imperfection, misalignment, and tolerances in the sensor’s mechanical design, which introduces challenging-to-model nonlinearities.

**Figure 6:**
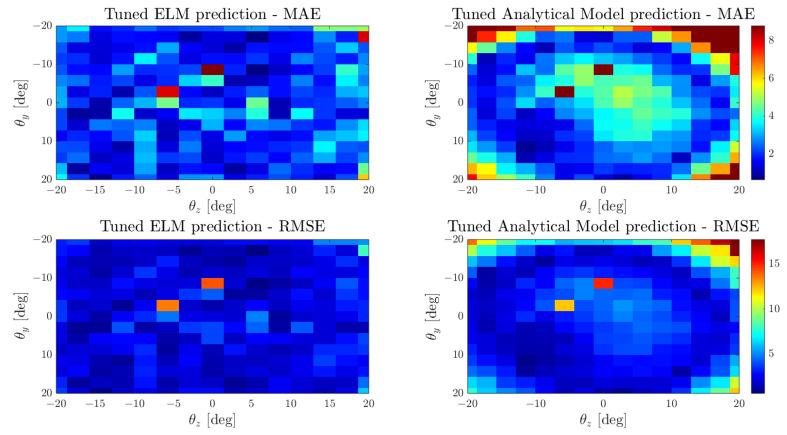
Mean average error and root mean square error comparison between the ELM and analytical models tuned for a single whisker.

As shown in Figure 6, a comparison is drawn between a finely-tuned analytical model and a specific ELM applied to a single-whisker dataset. The figure highlights the precision of these models within the robot’s captured workspace, presenting MAE and RMSE values for each cell. Upon observation, it becomes evident that the ELM performs well across the entire range of the whisker sensor, while the analytical model exhibits higher errors at higher rotational values. These findings affirm that the analytical model encounters challenges in predicting higher rotational angles, as nonlinearities are more likely to impact precision in this context.

In order to extend the model to cover the remaining whiskers, we opted to create a comprehensive general model for simplicity. This involved combining all the recorded whisker data into a single dataset. We then tuned and evaluated both analytical and data-driven models using these unified models. For the analytical model, we determined both bias and scaling factors by averaging the results presented in Table 2. Regarding the ELM model, we trained the new model using the combined whisker dataset. This time, we adopted a 70/30 split for training the ELM, where the total size of the dataset comprises 2094 recordings.

**Figure 7:**
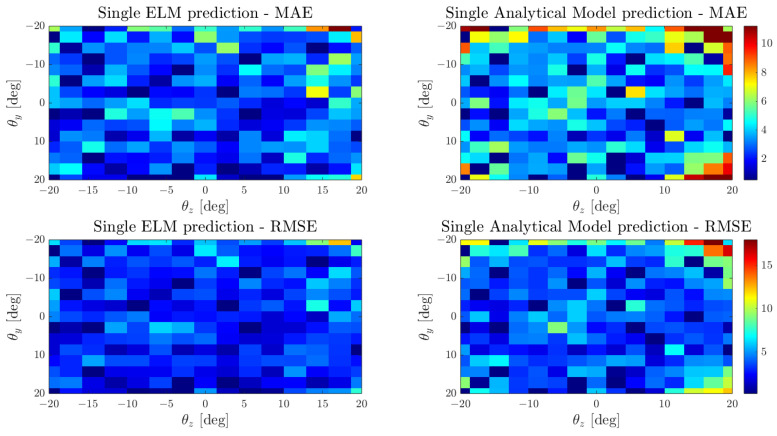
Mean average error and root mean square error comparison between the ELM and analytical models for the combined whisker dataset.

To validate these models, we again compared the prediction and speed of both models in the test set to ensure that the ELM was not trained with them. Table 4 and Figure 7 present the comparison of both models for the representation of the whisker workspace. As expected, the ELM model results in a more suitable model, since it presents lower errors and deviation across the combined whiskers workspace.

Consequently, the data-driven approach was chosen to ensure a faster sensor response given its superior precision and speed. The use of the same neural network trained with data from the collection of whiskers, along with appropriately scaled input values from other whiskers, was determined to yield relatively accurate results. However, to improve the accuracy and reliability of the model, each whisker sensor must undergo a specific tuning process to address imperfections that arise from the manufacturing process.

## 5. 3D-Mapping Implementation

**Figure 8:**
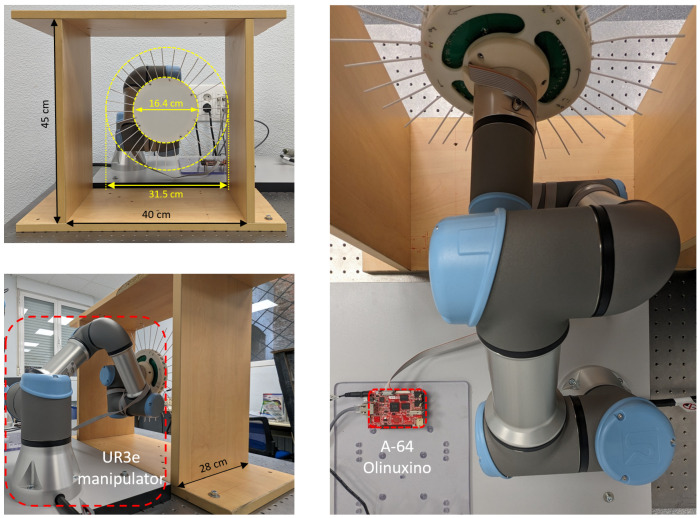
3D mapping testbed for environment reconstruction. The wood structure represents a simple section of a mining environment. The surfaces to be mapped are the inner faces of the wooden box.

After successfully validating the whisker model, we proceeded to implement it for a 3D-mapping scenario. In this occasion, the test platform, shown in Figure 8, incorporates the whisker disk as the end effector of the Universal Robots UR3e manipulator, while a rectangular box structure is securely attached to a technical table to mimic the basic geometry of a mine.

In order to visualize the point cloud produced by the whisker disk, we developed an application utilizing UR’s ROS2 driver package [30] and Rviz for visualization purposes. The disk was attached to the UR3e arm to determine its position and orientation in space. The whiskers were represented using a rigid bar featuring two rotational joints at the pivot point of the whisker, with each rotation angle determined by the whisker rotational model.

For reading the 3D Hall-effect sensors, an Olimex A64 computer running an Ubuntu image was programed to execute a routine that verified the proper performance of each sensor and then published the readings in an ROS2 topic. A custom message was created to transmit all 32 magnetic field measurements in a single packet. The topic publishing rate was fixed at 10 Hz.

To generate the point cloud, a node was written to receive the custom whisker message and convert it into the spatial position of the whisker tip. Before publishing the point cloud, a condition to filter out non-deflected whiskers was implemented. The condition specified that the sum of the squares of the rotation angles of the whisker must exceed a threshold to be included in the point cloud. The threshold was empirically determined as 0.09, which yielded the best results.

### 3D-Mapping Experiment

**Figure 9:**
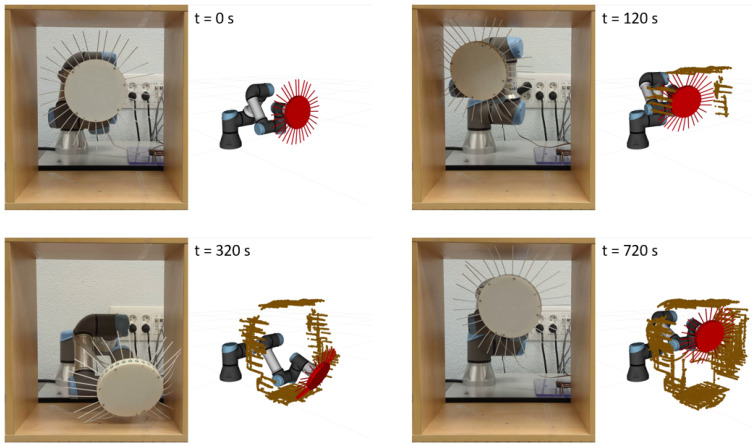
Environment reconstruction from the 3D-mapping experiment.

The mapping experiment was carried out by teleoperating the robot arm to ensure that the whiskers were in contact with the surface being mapped. Figure 9 and Video S1 highlight the generation of the point cloud based on deflected whiskers. This approach demonstrated good performance in determining the shape and approximate geometry of the environment.

**Figure 10:**
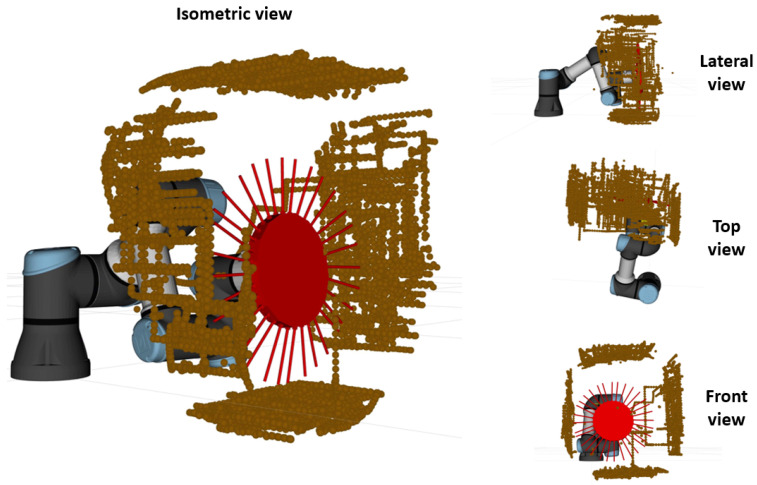
Orthographic views of the generated 3D pointcloud resulting from the mapping experiment.

The generated point cloud is shown in Figure 10. It reveals an accurate representation of the box geometry, even though it presents minor errors observed in the central section of the box. These errors may arise from deflections in the whiskers, causing them to bend to such a degree that they do not fully return to their nominal shape and are captured by the point cloud.

**Figure 11:**
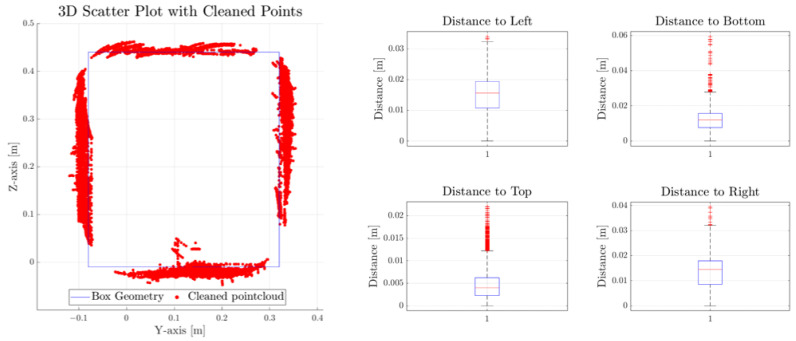
Three-dimensional point cloud comparison and error estimation between the ground truth and the prediction of each surface.

To quantify the accuracy of the 3D geometry prediction, we performed additional mapping experiments and exported and processed the resulting point cloud by applying a clustering algorithm and implementing a filter based on the Mahalanobis distance to eliminate outliers. The results of the refined point cloud are presented in Figure 11 and Table 5, which confirms the reliability of the proposed method with average errors close to millimeter precision on almost all surfaces. To obtain the distance difference from each surface, we projected the captured points onto the closest neighboring surface of the box and measured the Euclidean distance for each pair of points. Our analysis revealed that the predicted dimensions of the box measure 42.74 cm × 46.42 cm × 29.11 cm, showing a good approximation from the actual dimensions of the box (40 cm × 45 cm × 28 cm).

Finally, it should be noted that the presented solution has been designed to work as a robotic module that can be adapted to the scaled-down version of the robominer platform [21], to give the robot the ability to map unstructured environments while operating. However, this concept can be easily adapted to larger mine-ready platforms.

## 6. Conclusions

This study presented the design, modeling, and implementation of a sensor disk composed of a circular array of 32 whisker sensors for 3D mapping in unstructured environments. The design of the whisker comprises a plastic piece called a magnet holder, where a polystyrene rod and N52 permanent magnet are attached, and a 3D Hall-effect sensor that measures the magnetic flux generated by the magnet. The magnet holder features a spherical contact that acts as a ball joint. Each magnet holder is housed inside a silicone structure, and the magnetic sensors are installed on a single PCB.

The whisker was modeled following both analytical and data-driven approaches. For the analytical model, a series of relationships between the rotation angles and the magnetic flux measurements were derived based on the sensor manufacturer recommendations. To account for misalignment and variability from whisker to whisker, scaling and bias factors were included into the model and tuned based on experimental data, following a nonlinear least squares method. For the data collection process, a testbed featuring a UR3e robot was built to record the rotations of the whisker and the sensor measurements of the magnetic sensor.

For the data-driven approach, first a model selection process determined which algorithm resulted in the best performance considering both the precision and prediction time. Four widely used algorithms were considered, and we finally selected the ELM, as it presented the best balance between speed and accuracy.

To evaluate the performance of the models on the different sensors, six different whiskers were evaluated for both models. When comparing the results of the models, it was determined that the data-driven approach resulted in a more robust and faster performance than the analytical model, since it can account for nonlinearities that are not present in the analytical model. To account for the remaining sensors, a general model was developed and tested for both approaches, where the data captured from the recorded whiskers were merged into a single dataset. After tuning and evaluating the model, it was determined that the data-driven approach resulted in a more precise and robust solution. In addition, it was observed that the analytical model had difficulties estimating the rotational angles for large deflections of the whisker sensor, due to the appearance of difficult-to-model nonlinearities that are not considered. It is worth noting that to achieve optimal performance, each whisker must undergo an individual tuning process.

Once the whisker model was validated, the sensor disk was mounted in a serial manipulator to perform 3D-mapping experiments. A second testbed included a UR3e manipulator and a rectangular wood box that resembles a simple section of a mine geometry. The robot arm was teleoperated to map the inner surface of the rectangular box, resulting in a successful mapping of the geometry, thus validating the sensor capacity to reconstruct 3D environments. Although the sensing device was tested on a robot manipulator, the simple and easily adaptable design can be mounted on different robotic platforms, including mobile robots.

Recommendations for future developments include improvements to the mechanical design of the whisker to ensure its return to its nominal shape after bending as well as the design of new shapes of the whisker sensor to mimic the mole rat snout to improve the mapping performance in tight corners. Further advancements should also focus on refining the model to accurately identify contact points along the whisker, particularly in scenarios involving complex surface interactions, thus enhancing the overall accuracy and applicability of the system. Other studies can be carried out to generate a smart autonomous mapping algorithm that optimizes the mapping procedure.

## Figures and Tables

**Table 1:** Machine learning algorithms comparison.

Whisker Rotation	ELM (N_hidden_neurons = 30)			SVR (C = 8, Epsilon = 0.11)			MLP Single Layer (N_hidden_neurons = 100)			KNN-R (N_neighbors = 3)		
	MAE [rad]	RMSE [rad]	Time [ms]	MAE [rad]	RMSE [rad]	Time [ms]	MAE [rad]	RMSE [rad]	Time [ms]	MAE [rad]	RMSE [rad]	Time [ms]
$\theta_{z}$	0.0221	0.0273	0.1	0.0453	0.0529	1.14	0.0286	0.0364	0.37	0.0155	0.0205	1.05
$\theta_{y}$	0.0364	0.0465		0.0512	0.0609		0.0447	0.0609		0.0230	0.0315	

**Table 2:** Calibration for the analytical whisker rotational model.

Whisker	Model Parameters				$\theta_{z}$			$\theta_{y}$			Time [ms]
	${\mathrm{sc}}_{\theta_{z}}$	$b_{\theta_{z}}$ [rad]	${\mathrm{sc}}_{\theta_{y}}$	$b_{\theta_{y}}$ [rad]	MAE [rad]	RMSE [rad]	$R^{2}$	MAE [rad]	RMSE [rad]	$R^{2}$	
$w_{1}$	2.24	−0.0533	1.66	−0.0228	0.036	0.0489	0.945	0.0514	0.0723	0.8801	0.85
$w_{2}$	2.44	0.001	1.9	−0.04	0.0352	0.0469	0.9513	0.0577	0.0736	0.8681	0.72
$w_{3}$	2.27	−0.0321	1.56	0.01	0.0355	0.0477	0.949	0.052	0.0676	0.8973	0.64
$w_{4}$	2.65	0.054	2.12	0.05	0.0507	0.0702	0.8914	0.0818	0.1057	0.765	0.72
$w_{5}$	2.54	0.02	2	−0.01	0.0457	0.063	0.9157	0.0593	0.0829	0.8464	0.73
$w_{6}$	1.97	0.0476	1.5	−0.0834	0.036	0.0489	0.9468	0.0417	0.0557	0.931	0.73

**Table 3:** Calibration for the data driven whisker rotational model.

Whisker	$\theta_{z}$			$\theta_{y}$			Time [ms]
	MAE [rad]	RMSE [rad]	$R^{2}$	MAE [rad]	RMSE [rad]	$R^{2}$	
$w_{1}$	0.040	0.055	0.93	0.024	0.033	0.97	0.46
$w_{2}$	0.037	0.048	0.94	0.028	0.036	0.97	0.28
$w_{3}$	0.034	0.042	0.96	0.024	0.030	0.98	0.40
$w_{4}$	0.048	0.060	0.92	0.028	0.039	0.97	0.47
$w_{5}$	0.049	0.064	0.91	0.029	0.038	0.97	0.35
$w_{6}$	0.035	0.046	0.95	0.026	0.033	0.98	0.39

**Table 4:** Whisker rotation model comparison for general models for the combined whisker dataset.

Whisker Rotation Angles	Single ELM (N_hidden_neurons = 30)				Single Analytical Model (${\mathrm{sc}}_{\theta_{z}}$ = 2.37, ${\mathrm{sc}}_{\theta_{y}}$ = 1.75 $b_{\theta_{z}}$ = −0.01, $b_{\theta_{y}}$ = −0.02 )			
	MAE [rad]	RMSE [rad]	$R^{2}$	Time [ms]	MAE [rad]	RMSE [rad]	$R^{2}$	Time [ms]
$\theta_{z}$	0.0352	0.0464	0.95	0.18	0.0505	0.0665	0.90	0.87
$\theta_{y}$	0.0388	0.0537	0.94		0.0589	0.0833	0.84	

**Table 5:** Maximum and average errors derived from the post-processed point cloud and the projected points onto the adjacent box surface.

Box Inner Surface	Max Error [m]	Mean Error [m]
Left	0.0339	0.0149
Right	0.0394	0.0132
Top	0.022	0.0052
Bottom	0.0593	0.0123

## Data Availability

The data captured for the whisker sensors are available in the following repo: https://github.com/virgilio96upm/whisker-modeling/tree/main (accessed on 31 December 2023).

## Supplementary Materials

The following supporting information can be downloaded at: https://zenodo.org/records/10447310 (accessed on 31 December 2023), Video S1: whisker_demo.mp4.
